# Investigation of Volatile Iridoid Terpenes in *Nepeta cataria* L. (Catnip) Genotypes

**DOI:** 10.3390/molecules27207057

**Published:** 2022-10-19

**Authors:** Harna Patel, Erik Nunes Gomes, Bo Yuan, Weiting Lyu, Qingli Wu, James E. Simon

**Affiliations:** 1 New Use Agriculture and Natural Plant Products Program, Department of Plant Biology, Rutgers University, New Brunswick, NJ 08901, USA; 2 CAPES Foundation, Ministry of Education of Brazil, Brasilia 70.040-020, DF, Brazil; 3 Department of Medicinal Chemistry, Ernest Mario School of Pharmacy, Rutgers University, Piscataway, NJ 08854, USA; 4 Center for Agricultural Food Ecosystems, Institute of Food, Nutrition & Health, Rutgers University, 61 Dudley Rd., New Brunswick, NJ 08901, USA

**Keywords:** arthropod repellent, chemodiversity, dihydronepetalactone, germplasm, Lamiaceae, nepetalactam, nepetalic acid

## Abstract

Catnip (*Nepeta cataria* L.) is of scientific interest largely due to the production of nepetalactones, volatile iridoid terpenes with strong arthropod repellent activity. However, the plant can also produce other bioactive volatile iridoids, such as nepetalic acid (NA), nepetalactam (NT) and dihydronepetalactone (DHNL) that have not been studied extensively. Germplasm studies on plants that can produce such compounds are scarce. The present study evaluated the chemical diversity of catnip genotypes with a focus on NA, NT and DHNL. A total of 34 genotypes were harvested at different times over two years. The ethanolic extract of the plants was screened for iridoids by ultra-high-performance liquid chromatography/triple quadrupole mass spectrometry. CR9 × CR3 genotype had the highest value for biomass yield, while cultivar CR9 had the highest value for accumulated NA. Genotype UK.2 had the highest value for accumulated NT yield and CR5 had the highest value for accumulated DHNL. Overall, patented cultivars and elite selections performed better than other less studied genotypes. Harvest time influenced the accumulation of secondary metabolites differentially for the genotypes. This is the first germplasm study with a focus on these iridoid compounds, yet more studies are necessary as genotype characterization is essential for breeding and standardization of products for industry.

## 1. Introduction

Since ancient times, plants have been used as food and medicine [1]. People have been incorporating the secondary metabolites found in plants in their daily lives not only for flavoring and preservatives, but also for treatment of various ailments [2]. Catnip (*Nepeta cataria* L.), belonging to the Lamiaceae family is famous for its euphoric effect on domestic cats and possesses valuable secondary metabolites with medicinal properties. *N. cataria* is also the most studied species of the genus *Nepeta* [2,3,4]. Historically, catnip has been used in infusions, tinctures, teas, juices, and poultices [5]. Due to its lemony mint flavor, catnip has been frequently used in cooking as well [6]. Catnip’s fresh or dried leaves and flowers are used to make soup, sauce and cheese in Iran and a few other countries [7]. In addition to being used in cooking, catnip has been widely used in traditional medicine as a folk remedy for cough and cold, colic, asthma, and diarrhea [6,8]. Catnip also possesses antimicrobial and antioxidant activities [9,10]. 

Catnip contains many secondary metabolites, including nepetalactone, a bicyclic monoterpene, belonging to the class of chemical compounds known as iridoids [11,12]. In addition to causing euphoria in cats, nepetalactone is an insect repellent [11,13]. Other less studied iridoid compounds such as nepetalic acid, nepetalactam and dihydronepetalactone have been reported to be found in catnip [14,15]. Nepetalic acid is formed via hydrolysis of nepetalactone [16]. Nepetalactam, a nitrogen analog of nepetalactone, can be prepared from nepetalic acid and nepetalactone [17]. Dihydronepetalactone is formed by hydrogenating nepetalactone [14]. Although literature is scarce regarding the bioactivity of nepetalic acid, a report by Harney et al. [18], shows that this iridoid along with nepetalactone and catnip essential oil has a depressant effect in mice when administered intraperitoneally [18]. Nepetalactam has shown bioactivity against *Aedes aegypti* mosquitoes in a feeding deterrence assay [15]. Dihydronepetalactone has also been reported to be a safe and effective topical insect repellent [19]. Due to the presence of secondary metabolites with insect repellent activity, catnip can be a commercially valuable plant. 

Opportunities to cultivate medicinal herbs in the USA have increased due to the expansion of the market for high value niche crops [20]. The opportunity for commercialization of catnip for producing mosquito repellent is apparent as the mosquito repellent market has the potential to grow by USD 3.60 billion during 2021–2025, with Asia-Pacific and Middle East and Africa being some of the fastest growing regions with an increasing demand for mosquito repellents [21]. Areas in sub–Saharan Africa where the afro tropical mosquito is a major pathogen vector for life threatening diseases such as malaria, growing catnip for producing insect repellents can be an excellent alternative to hard to purchase and/or expensive synthetic repellents such as DEET and it may be safer and just as effective [22,23,24,25,26]. In addition, growing catnip can provide opportunities for additional income for rural communities and help stimulate the local economy [11,27]. Infectious diseases involving vector borne pathogens that impact humans and livestock pose a global threat as they do not adhere to human-made borders, an issue that may be worsened by the changing climate [28]. To mitigate and prevent further damage caused to human life and livestock by vector borne pathogens, finding, and implementing new methods of controlling them is necessary [28]. In addition to the risk faced by humans and livestock due to vector borne pathogens, the plants with the secondary metabolites are at risk themselves from the changing climate. Medicinal and aromatic plants (MAPs) are particularly valuable as they are economically important in addition to being used in traditional medicine systems [29]. 

In this context, plant genetic diversity can be an important tool in helping address these changes because it can serve as a source of novel traits that may help tolerate biotic and abiotic stresses in plants [30]. Characterizing the genetic diversity of the germplasm is a vital part of improving crops [31]. Genetic diversity in a germplasm can be utilized by plant breeders to develop and improve cultivars with desirable traits such as high yield, increased pest, and disease resistance and more [32]. Thus, identifying diverse lines or creating diversity within an existing germplasm are the major goals of crop improvement programs [30]. 

The few breeding and genetic studies done on catnip confirm that *N. cataria* is an orphan crop whose commercially available varieties are more characteristic of landraces rather than genetically improved cultivated varieties [11,12]. To address this issue, our team has developed two catnip cultivars ‘CR3′ and ‘CR9′ for production in North America with improved traits such as vertical growing habit that allows for mechanical harvest, increased essential oil yield along with higher levels of nepetalactone isomers E,Z-nepetalactone and Z,E-nepetalactone, respectively, in their essential oil compared to commercial genotypes [33,34]. Since the production of secondary metabolites is highly sensitive to environmental changes, it is important to consider the effect of such variables on the productivity of different genotypes [11]. Thus, the current study aims to continue the work of developing and improving catnip germplasm by assessing the effect of genotype, harvest time and their interaction on the biomass yield and content of less studied iridoid terpene metabolites in 34 genotypes of *N. cataria.*

## 2. Results and Discussion

Following analysis of variance, a genotype effect significant at *p* ≤ 0.01 was observed for biomass, nepetalic acid (NA), nepetalactam (NT) and dihydronepetalactone (DHNL) contents. The harvest effect was significant at *p* ≤ 0.05 for biomass and at *p* ≤ 0.01 for NA, NT, and DHNL. Additionally, the interaction effect between genotype and harvest was significant for biomass, NA, and NT (*p* ≤ 0.01) and DHNL (*p* ≤ 0.05) (Table 1). The unfolding of the isolated effects and interactions are described in the following paragraphs.

### 2.1. Genotype Effect

For biomass, on average, genotypes UK.5 and UK.9 had the worst performance, followed by genotypes C246, CIT, CL2, CR2.3, G1, UK.6 and UK.17. The other genotypes, with a superior performance, varied from 107.9 to 155.6 g of dry biomass per plant but did not differ statistically from each other (Table 2). 

In the average of 3 harvests, the CR9 × CR3 genotype, a cross between the two commercialized cultivated varieties CR9 and CR3 had the highest value of the biomass yield at 155.6 g/plant while, UK.9, a previously unexplored genotype of catnip had the lowest average biomass yield at 59.8 g/plant which was statistically significantly lower compared to all the genotypes tested over a two-year period (Table 2).

Previous studies on CR3 and CR9 have shown that those cultivars present biomass productivity that is superior to other commercial genotypes [33,34]. Our results show that plants that originated from the crossing of CR3 and CR9 inherit their biomass accumulations, a significant trait that can be exploited commercially. The variations observed on biomass accumulation on the genotypes, reinforce the necessity of further studies on catnip germplasm with quantitative and qualitative focuses (i.e., total productivity and chemical composition) and those studies should be standardized in terms of seasonality as it profoundly influences primary and secondary metabolites accumulation [35,36].

For Nepetalic Acid (NA), CR9 was the highest producing genotype in the average of all harvests at 863.6 mg/100 g of dry biomass. UK.9, CR5 and CR9 × CR3 were second best on average at producing NA with a range of 728.8 mg/100 g–660.0 mg/100 g and no statistical difference between the three. CL1 had the lowest NA content at 11.9 mg/100 g and was statistically different from CR9, UK.9, CR5 and CR9 × CR3 (Table 2). In addition to *N. cataria*, NA has also been reported to be found in *Nepeta nuda*, *Nepeta nuda* ssp. *albiflora*, and *Nepeta caesarea* among others [37,38,39]. Biological studies demonstrated that NA, along with other iridoid terpenes, has a depressant effect in mice when administered intraperitoneally [18]. This experiment demonstrated that these compounds could impact the behavior of animals other than insects and felines [18]. An additional application for this compound can be derived from the fact that it can be synthetically converted to nepetalactone [40]. This makes nepetalic acid and nepetalic acid-producing plants potentially valuable industrial resources.

The average yield of nepetalactam (NT) was much lower compared to that of nepetalic acid (NA). Genotype UK.2 had the highest content of NT at 25.5 mg/100 g, followed by the CR genotypes with CR9 and CR1 having the second highest amount at 19.1 mg/100 g followed by CR5, CR2.3, CR2, and CR3 ranging from 13.9 mg/100 g–16.9 mg/100 g. The commercial genotype CIT had the lowest content of NT at 2.8 mg/100 g (Table 2).

Nepetalactam was first tentatively identified from the genus *Moluccella*, belonging to the same family as the genus *Nepeta*, the Lamiaceae family [41]. This compound has been previously reported as a component of the essential oil of *Nepeta cataria*, [37] and has shown to act as an antagonist by inhibiting odorant receptors AaOR2, AaOR7 and AaOR8 in *Aedes aegypti* [42]. In addition to being produced in plants, nepetalactam can also be synthesized from nepetalactone as demonstrated by Chauhan et al. [15] who successfully produced nepetalactam in a two-step synthesis reaction. Nepetalactam and its analogues have been tested in a feeding deterrence assay using *Aedes aegypti* mosquitoes showing repellency comparable to that of DEET. The results showed that the synthesized compounds have comparable activity to DEET, repellent’s gold standard [15].

The results from the feeding deterrence assay indicate that nepetalactam should be tested further and against other species of arthropods as well [15]. Formulations can help stabilize the compounds and ensure that the bioactivity of the compound is retained as demonstrated in other compounds from the Lamiaceae family [43]. The potential use of nepetalactam as an insect repellent adds to the agronomic/breeding value of catnip.

For dihydronepetalactone (DHNL), on average genotype CR5 had the highest value at 7.9 mg/100 g followed by CR2, CR2.3, CR1, CR9, UK.1 and UK.9. CR2, CR2.3, CR1, CR9, UK.1 and UK.9 had the second highest values of DHNL ranging from 6.5 mg/100 g to 5.3 mg/100 g and were statistically equal. C245 had the lowest content of DHNL at 3.4 mg/100 g (Table 2). 

Similar to nepetalic acid and nepetalactam, dihydronepetalactone is also reported as a constituent of *N. cataria* essential oil as an important aromatic component [19,37]. Like nepetalactone, the major constituent of catnip oil, DHNL is a potent and relatively safe insect repellent, having shown repellency against mosquitoes (*Aedes aegypti* L., *Anopheles albimanus* Wiedemann and *Aedes intrudens* Dyar), black flies (*Simulium decorum* Walker), stable flies (*Stomoxys calcitrans* L) and deer tick (*Ixodes scapularis* Say) nymphs [14,19,44]. In experiments involving human subjects, DHNL provided excellent repellency against *Anopheles albimanus* mosquito bites for up to 5 h, providing repellency that was statistically the same as DEET [14].

### 2.2. Harvest Effect

Out of the 3 harvests, harvest 1 (summer) and harvest 2 (fall) in the year 2017 had the highest biomass with no statistically significant difference between them. The 2018 harvest (summer) had the lowest value for biomass yield out of all 3 (Table 3). 

For nepetalic acid (NA), the 2018 harvest (summer) had the highest amount followed by 2017 harvest 1 and harvest 2 (summer and fall). All results were statistically different from each other (Table 3). Variation on secondary metabolites in different seasons can be attributed to environmental conditions such as temperature, rainfall, humidity, etc. The precipitation, temperature and humidity were higher at the time of the first harvest in July 2017, compared to the first and only harvest in June 2018. In *Nepeta cataria*, the ratio of the nepetalactone isomers (Z, E- and E, Z-nepetalactone) has been reported to be affected by seasonal changes when measured on a weekly basis [36] and, as nepetalic acid can be derived from nepetalactone [40], it is possible that it is influenced by similar factors.

The amount of nepetalactam (NT) was the highest in 2017 harvest 1 (summer) while 2017 harvest 2 and the 2018 harvest (fall and summer) did not differ statistically from each other (Table 3).

Harvest 1 of 2017 (summer) had the highest yield of dihydronepetalactone (DHNL) followed by the harvest of 2018 (summer) and harvest 2 of 2017 (fall). The summer season is shown to have higher content of DHNL compared to the fall season, thus variables such as temperature, precipitation and humidity which differ between seasons can be factors that also affect the content of DHNL.

Chemical composition can be influenced by seasonal variation as shown by Chaves et al. in their study looking at two Brazilian medicinal plants *P. marginatum* and *G. gracilliflora*. *P. marginatum* contained higher concentrations of polyphenols in the winter while *G. gracilliflora* had higher concentration of polyphenols in the summer. For flavonoids, the opposite trend was observed [45]. Other plants in the Lamiaceae or mint family such as basil (*Ocimum basilicum*) have also displayed variation in the essential oil content as well as its chemical constituents in winter vs. summer [46]. The samples collected in the summer were richer in sesquiterpene hydrocarbons (24.3%) vs. the ones collected in the winter which were richer in oxygenated monoterpenes (68.9%) [46]. 

### 2.3. Genotype and Harvest Interaction

#### 2.3.1. Biomass

**Figure 1:**
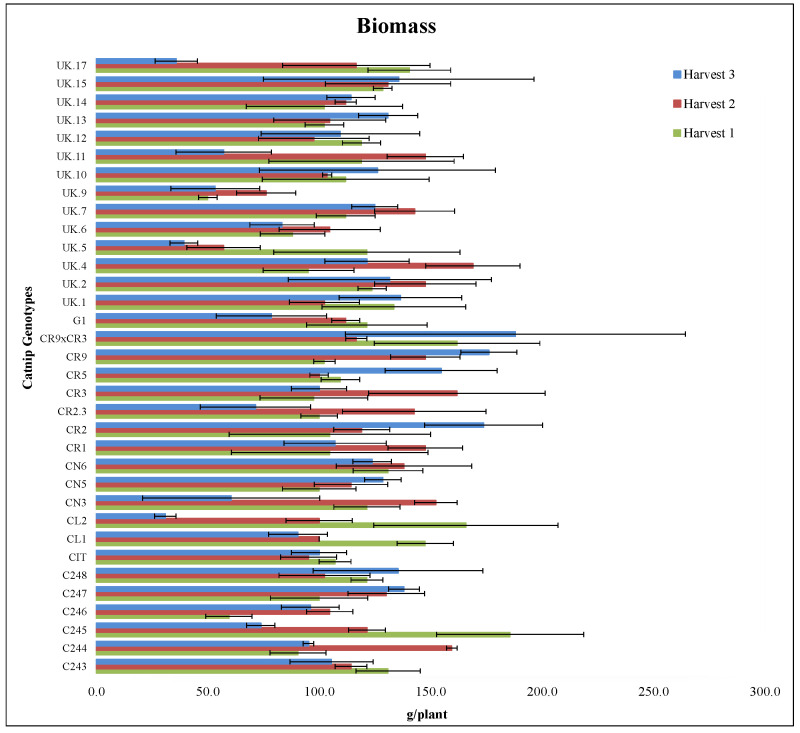
Dry biomass (g/plant) of catnip genotypes at three different harvest times. Pittstown, NJ, USA, 2017 and 2018. Error bars represent standard errors. Harvest 1 = July 2017, Harvest 2 = September 2017, Harvest 3 = June 2018. Detailed statistical analyses of the interaction effects are presented in the Supplementary Material Table S1.

For the biomass yield, in 2017 harvest 1, all the genotypes were statistically equal (Figure 1, Table S1). However, differences occurred in 2017 harvest 2 and the 2018 harvest. For 2017 harvest 2, genotypes C244, C247, CN3, CN6, CR1, CR2.3, CR3, CR9, UK.2, UK.5 UK.7, UK.11, UK.15 had the best performance (not differing among themselves). Furthermore, for plants harvested in 2018, C244, C245, C246, CIT, CL1, CL2, CN3, CR2.3, G1, UK.5, UK.6, UK.9, UK.11, UK.17 had the worst performance (Figure 1, Table S1).

As for some of the elite lines from Rutgers’s breeding program and patented cultivars, CR5, CR9 × CR3, CL1, CN5, CN6, CR1, and CR2.3 did not vary statistically across harvests. In contrast, CR9 accumulated lower biomass in the first harvest compared to the last two in which its performance improved. CR3 had the best performance in 2017 harvest 2 (Figure 1, Table S1). Considering that different genotypes may vary regarding their photosynthetic capabilities, it is expected that the environment may cause changes in biomass accumulation in a genotype-specific manner. Such interactions have been previously described in aromatic plants such as peppermint and spearmints [47], basil [48] and oregano [49].

#### 2.3.2. Nepetalic Acid

**Figure 2:**
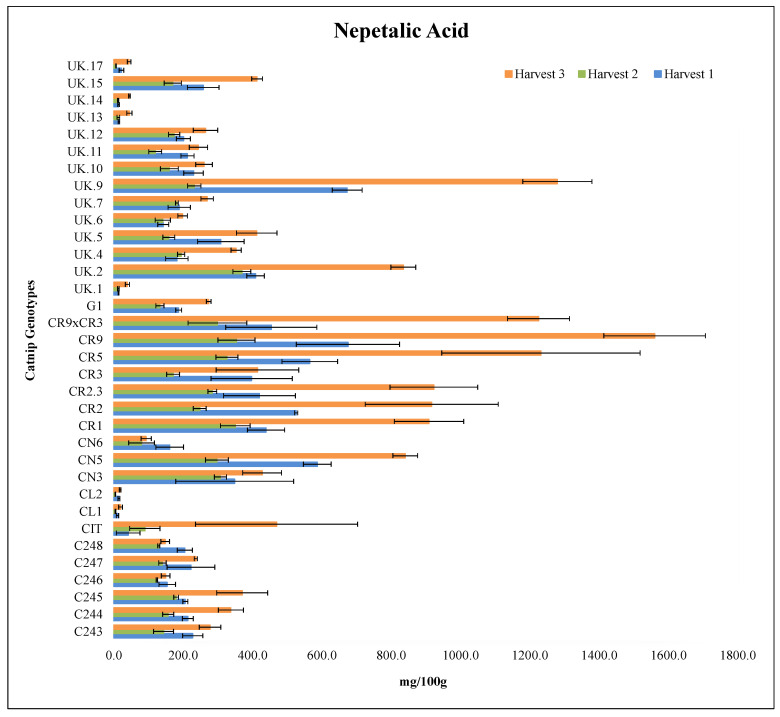
Nepetalic Acid (NA) content (mg/100 g) of catnip genotypes at three different harvest times. Pittstown NJ, USA, 2017 and 2018. Error bars represent standard errors. Harvest 1 = July 2017, Harvest 2 = September 2017, Harvest 3 = June 2018. Detailed statistical analyses of the interaction effects are presented in the Supplementary material Table S2.

In 2017 harvest 1, CR9 contained the highest content of NA at 675.9 mg/plant while CL1 yielded the lowest value of NA at 10.8 mg/plant. CR9 was followed by UK.9, CN5, CR5 and CR2 in terms of biomass, with no statistical difference among themselves. CR9 × CR3, CR3, CR1, CR2.3, CN3 and UK.2 follow suit being the second best and statistically equal to each other (Figure 2, Table S2).

UK.2 had the highest NA value at 369.7 mg/100 g (not statistically different from CN3, CN5, CR1, CR2, CR2.3, CR5, CR9, CR9 × CR3, and UK.9) in 2017 harvest 2. CN5, CR2, CR5 and CR9 and UK.9 were statistically equal while CR3 and CN6 were statistically different. The other genotypes had a worse performance in 2017 harvest 2 (not differing among themselves) (Figure 2, Table S2).

In the 2018 harvest, cultivar CR9 had the single highest NA value at 1560.9 mg/100 g, followed by UK.9 (1280.4 mg/100 g), CR5 (1233.0 mg/100 g) and CR9 × CR3 (1226.1 mg/100 g). CL2 had the lowest value of NA in this harvest at 18.1 mg/100 g like it did in 2017 harvest 2 as well (not statistically different from C246, C248, CL1, CN6, UK.1, UK.6, UK.13 and UK.17) (Figure 2, Table S2).

Variations in secondary metabolites in different harvests is a well-studied phenomenon, occurring in multiple species of the family Lamiaceae [47,50,51,52,53,54]. Although no reports of such events could be found for nepetalic acid, nepetalactones, compounds that are structurally similar to nepetalic acid, have been studied in this regard [36]. In *Nepeta cataria*, the ratio of the nepetalactone isomers (Z, E- and E, Z-nepetalactone) has been affected by seasonal changes when measured on a weekly basis [36]. In a study conducted in Giza, Egypt by Said Al-Ahl et al., [55], the chemical composition of the catnip (*N. cataria*) and lemon catnip (*N. cataria* var. *citriodora*) essential oil varied based on the time of harvest as well as the chemotype of the plants. While the essential oil of the *N. cataria* plant is dominated by nepetalactone isomers, other compounds such as α-humulene, α-pinene, D-limonene, sabinene, caryophyllene oxide, citronellol, geraniol, thymol, *trans*-caryophyllene have also been reported [55]. The composition of catnip essential oil has shown to vary widely depending on the environment, season and geography [56].

#### 2.3.3. Nepetalactam

**Figure 3:**
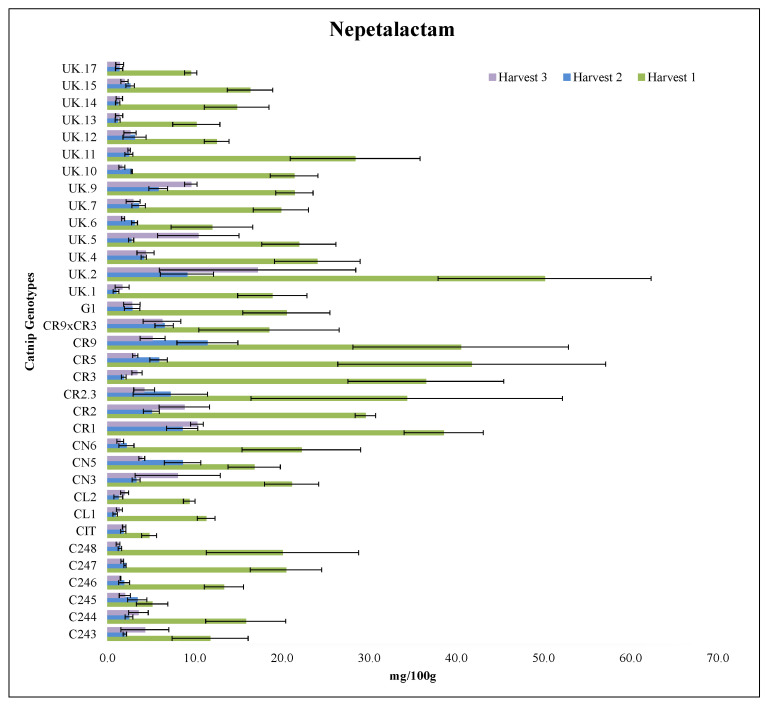
Nepetalactam (NT) content (mg/100g) of catnip genotypes at three different harvest times. Pittstown NJ, United States, 2017 and 2018. Error bars represent standard errors. Harvest 1 = July 2017, Harvest 2 = September 2017, Harvest 3 = June 2018. Detailed statistical analyses of the interaction effects are presented in the Supplementary material Table S3.

For NT content, in 2017 harvest 1, UK.2, CR5, CR9 and CR1 had the highest values ranging between 38.6 mg/100 g–50.2 mg/100 g with no statistical difference among them. CR3, CR2.3 and CR2 had the second highest set of values ranging from 29.6 mg/100 g–36.5 mg/100 g and were also statistically equal to each other. CIT had the lowest value at 4.8 mg/100 g (Figure 3, Table S3). 

In 2017 harvest 2 and 2018, the genotypes did not differ statistically regarding nepetalactam content (Figure 3, Table S3). The CR genotypes (CR1, CR2, CR2.3M CR3, CR5, CR9, CR9 × CR3) and the CN genotypes (CN5 and CN6), had the highest values of NT in 2017 harvest 1 when comparing each genotype individually across all 3 harvests (Figure 3, Table S3). 

Nepetalactam, an iridoid monoterpene derived from nepetalactone, has shown insect repellent potential in previous studies, including modulatory function of mosquitoes’ olfactory receptors [15,42]. To the best of our knowledge, this is the first time that levels of nepetalactam are being reported to be influenced by the interaction between harvest times and genotypes. It is not clear if this compound occurs naturally in the plants or if it is produced by post-harvest handling procedures affecting nepetalactone. Future studies should address this aspect. However, the presence of this molecule in catnip tissues can become an interesting breeding trait, considering the important biological activities attributed to this compound.

#### 2.3.4. Dihydronepetalactone

**Figure 4:**
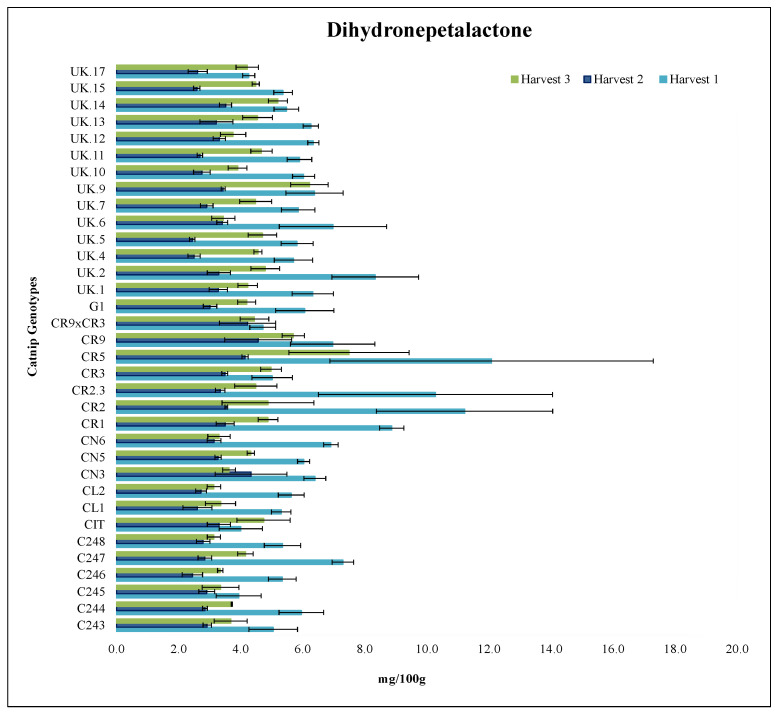
Dihydronepetalactone (DHNL) content (mg/100 g) of catnip genotypes at three different harvest times. Pittstown, NJ, USA, 2017 and 2018. Error bars represent standard error. Harvest 1 = July 2017, Harvest 2 = September 2017, Harvest 3 = June 2018. De-tailed statistical analyses of the interaction effects are presented in the Supplementary material Table S4.

For dihydronepetalactone (DHNL), in harvest 1 of 2017, CR5, CR2 and CR2.3 had the highest values, all statistically equal, ranging from 10.3 mg/100 g–12.1 mg/100 g, followed by CR1 (8.9 mg/100 g) and UK.2 (8.3 mg/100 g). The other genotypes showed the lowest values, not statistically different from each other for harvest 2017-1. For harvests 2017-2 and 2018, no statistical difference was observed among genotypes for DHNL (Figure 4, Table S4). 

CN5, CR3, CR9 and CR9 × CR3 were statistically the same in terms of harvest time across all three harvests. This indicates that for DHNL, the harvest time has little to no effect on the content of the compound within these. CR5 was statistically different across all three harvests, with its 2017 harvest 1 having the highest value out of all 3 harvests for this genotype (Figure 4, Table S4).

In other aromatic plants such as basil, interaction of genotype and successive harvests have also shown to impact the secondary metabolism and aroma profile, like the three Genovese basil (*Ocimum basilicum*) cultivars grown for production of pesto [57]. The second harvest resulted in an increase in the fresh biomass (172%) and phenolic acid content (413%) while certain aromatic compounds increased and/or decreased depending on the cultivar [57]. 

The effect of harvest time on four cultivars of parsley (*Petroselinum crispum*) were studied for two seasons in Giza, Egypt [58]. The plants were harvested five times before flowering each season. The highest biomass was obtained after the 3rd harvest while the content of the essential oil gradually increased from the first harvest to the fifth harvest. The highest essential oil yield for all cultivars was obtained from the 3rd and 4th harvests [58].

The effect of two different harvest times on the essential oil content and phenolic composition of two cultivated accessions of Salvia (*Salvia fructose* Mill) were studied in Thessaloniki, Greece [59]. The results showed that in early summer, the essential oil content as well as the dominant compound in the oil profile, 1,8-cineole was in higher amounts, with the content of the oil increasing from spring to summer (April–August) while decreasing in the autumn (September–October). Significant differences between the two populations were observed during the different harvest times when the compounds in the essential oil were quantified. On the other hand, the content of phenolic acids varied largely from spring to autumn [59]. 

Similarly, to the trend observed for nepetalactam, genotypes only differed among themselves in harvest 2017 1 for DHNL. Although it is hard to isolate the factors that can explain such behavior, one of the possible explanations can be the highest temperatures observed in July 2017, when the first harvest took place. It is well known that plants utilize secondary metabolites as heat stress modulators [60,61] and that some secondary metabolites will only be expressed in such conditions. It is also possible that the higher temperatures also increased the activity of insects [62,63,64], which in turn stimulated the differential production of repellent compounds by the plants.

### 2.4. Estimate of Biomass, Nepetalic Acid, Nepetalactam, and Dihydronepetalactone Yields (Sum of 3 Harvests)

**Figure 5:**
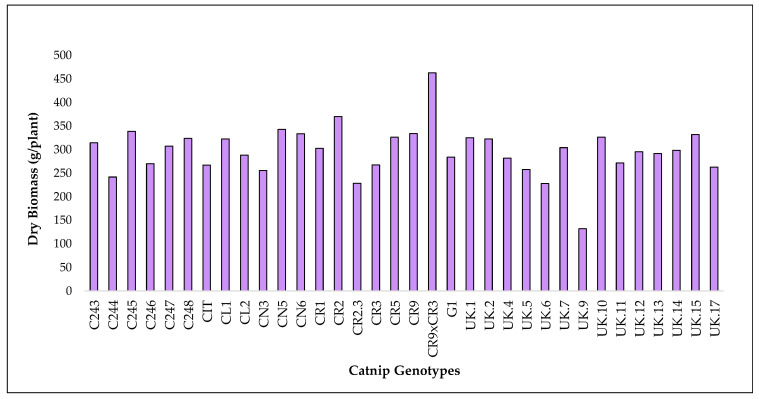
Accumulated biomass in different catnip genotypes after 3 successive harvests (2017 1 and 2 and 2018). Pittstown, NJ, USA.

Here, we present the accumulated values of biomass from 3 harvests. Such data can be used as an estimate of how much of each metabolite can be produced per cultivated area. Total accumulated biomass was calculated by the sum of the biomass of each genotype in each harvest (Figure 5, Table S5). 

Yield of specific metabolites was estimated by multiplying the content in mg per gram by the weight of the plants (g per plant) of each genotype. The accumulated yield of each metabolite was then obtained by the sum of the yields of each of the three harvests. 

As for the accumulated totals, genotype UK.9 had the lowest biomass yield at 78.6 g/plant and CR9 × CR3 had the highest biomass yield at 275.1 g/plant in 2017. In 2018, CL2 had the lowest and CR9 × CR3 had the highest at 31.0 g/plant and 188.1 g/plant respectively. Overall, when combining biomass from both years, UK.9 had the lowest at 131.9 g/plant and CR9 × CR3 had the highest at 463.2 g/plant out of all genotypes (Figure 5, Table S5).

**Figure 6:**
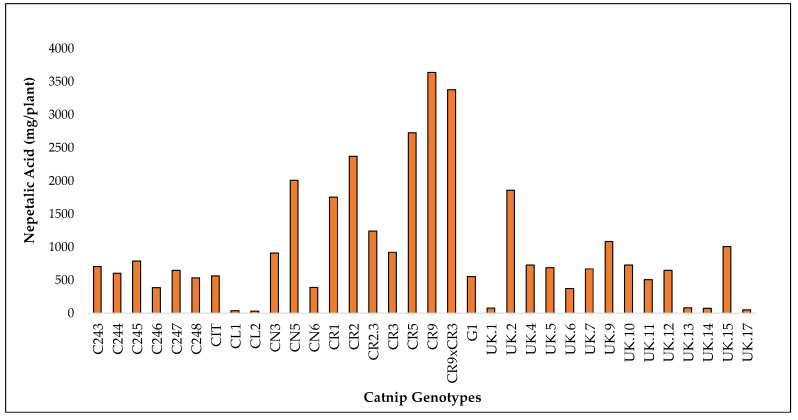
Accumulated yield of nepetalic acid in catnip genotypes after 3 successive harvests (2017 1 and 2 and 2018). Pittstown, NJ, USA.

Cultivar CR9 had the highest accumulated yield of NA over the two-year period. In 2017, CR9 had the highest yield at 887.8 mg/plant compared to CL1 which had the lowest at 20.2 mg/plant. In 2018, CL2 had the lowest NA yield at 5.6 mg/plant with CR9 having the highest at 2750.2 mg/plant. When combined, CR9 had the highest yield at 3638.0 mg/plant and CL2 had the lowest at 32.2 mg/plant at the end of three harvests. The NA content of CR9 increased overall from one year to the next (Figure 6, Table S5).

**Figure 7:**
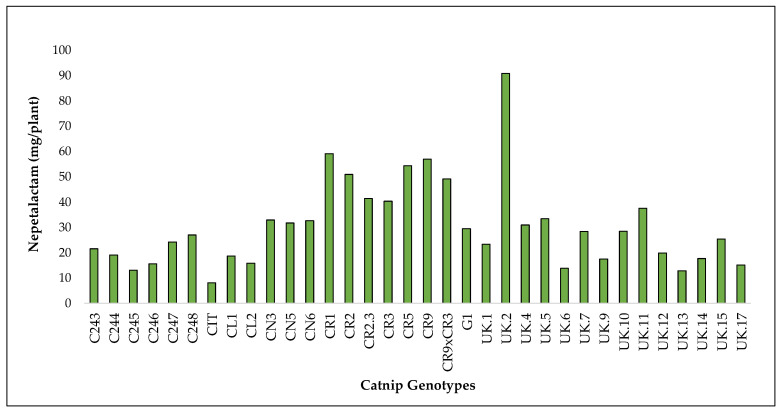
Accumulated yield of nepetalactam in catnip genotypes after 3 successive harvests (2017 1 and 2 and 2018). Pittstown, NJ, USA.

In terms of NT yield, CIT, the commercial genotype, had the lowest content at 6.2 mg/plant while UK.2 had the highest at 68.2 mg/plant in 2017. In 2018, UK.17 had the lowest at 0.5 mg/plant while UK.2 had the highest at 22.7 mg/plant. Overall, when combined, CIT had the lowest value at 8.1 mg/plant while UK.2 had the highest at 90.9 mg/plant. UK.2 had the highest NT levels across both years and when the yields were combined (Figure 7, Table S5). 

**Figure 8:**
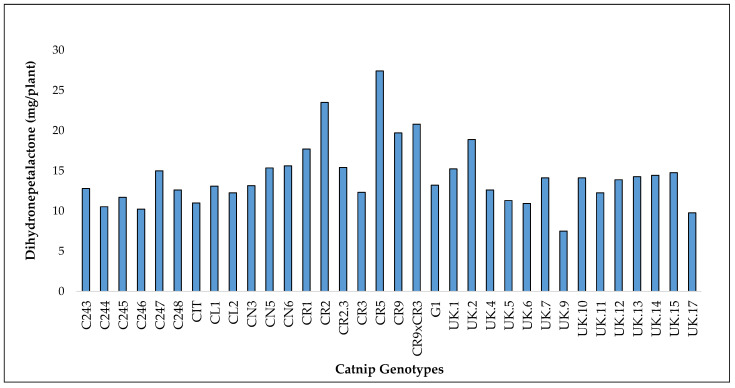
Accumulated yields of dihydronepetalactone in catnip genotypes after 3 successive harvests (2017 1 and 2 and 2018). Pittstown, NJ, USA.

In 2017, UK.9 had the lowest Dihydronepetalactone (DHNL) content at 4.2 mg/plant while CR5 had the highest at 15.8 mg/plant. In 2018, CL2 had the lowest DHNL at 1.0 mg/plant while CR5 had the highest DHNL at 11.6 mg/plant. (Figure 8, Table S5).

Combined, genotype UK9 had the lowest DHNL at 7.5 mg/plant while CR5 had the highest at 27.4 mg/plant. DHNL was the highest for 2018 and when the values were combined for both years. The observed values for NT and DHNL are lower than those of NA (Figure 8, Table S5).

**Figure 9:**
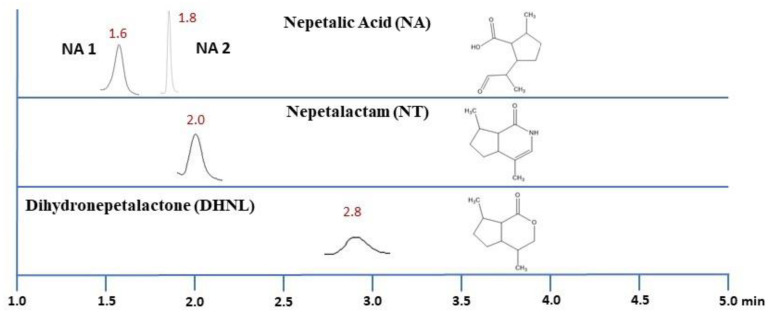
Representative chromatograms of Nepetalic Acid (NA), Nepetalactam (NT) and Dihydronepetalactone (DHNL) in CR3 by UHPLC-QqQ-MS/MS along with their chemical structures.

Overall CR9 × CR3 had the highest biomass (463.2 g/plant), CR9 had the highest NA (3638.0 mg/plant), UK.2 had the highest NT (90.0 mg/plant) and CR5 had the highest DHNL (27.4 mg/plant) (Figure 6, Figure 7, Figure 8 and Figure 9). Accumulated data shows interesting agronomic potential for CR9, CR9 × CR3 and CR5 for the production of high-value compounds for pharmaceutical purposes.

In previous studies, trends between harvest time and genotypes have been ob-served. Gomes et al. [35] conducted a study on the interaction between harvest times and genotypes of lemon catnips observed significant changes due to harvest times in a few of the lemon scented catnip lines. Statistically significant differences were observed between harvest times overall (average of 3 harvests) for each genotype as well [35]. Finally, the essential oil content showed an interaction effect between genotypes and harvest time with the commercial catnip genotype CIT displaying highest essential oil (0.75% in June 2018) in the third harvest and surpassing that of all other genotypes in all three harvests [35]. 

In addition to the aspects addressed in the present study, some of the other factors that are known to affect the total content and ratios of secondary metabolites in aromatic plants are rainfall, temperature, altitude, UV radiation, seasonality, circadian rhythm, plant development stage, attacks by herbivores and infections by pathogens to name a few [65]. In the future, studies can be carried out assess the effect of each of these variables either individually or together on the biomass yield and chemical composition of catnip to assist in breeding efforts to create new and improved cultivars.

## 3. Materials and Methods

### 3.1. Seedling Production and Transplantation

Catnip (*Nepeta cataria*) genotypes were selected from commercial sources and from the Rutgers University and New Jersey Agricultural Experiment Station germplasm collection and breeding program to evaluate the diversity in their chemical profiles. In this study, a commercial lemon catnip line, CIT (*Nepeta cataria* var. *citriodora*, from Jelitto Perennial Seeds, Louisville, KY, USA), plus two cultivars of catnip CR3 and CR9 developed for use in North America [33,34] were used. In addition, two elite lines (CR5 and CR9 × CR3), selections from the CR breeding line (CR1, CR2, and CR2.3), and lemon catnip selections (CL1, CL2, CN3, CN5, and CN6) [33,35] were also used. Lastly, the following genotypes C244, C245, C246, C247, C248, G1, UK.1, UK.2, UK.4, UK.5, UK.6, UK.7, UK.9, UK.10, UK.11, UK.12, UK.13, UK.14, UK.15, and UK.17 which have not been studied previously were also added to this experiment.

For seedling production, 128 cell plug trays were filled with commercial soil (Sun Gro Horticulture, Anderson, SC, USA). The genotypes were self-pollinated and the seeds resulting from this process were used to produce the propagules for the experiment, along with the seeds of the commercial genotype CIT. Seeds were sown into the trays and watered twice a day for 45 days until the plants reached transplanting size (15–20 cm tall). The seedlings were transplanted into the field in late May (2017) at the New Jersey Agricultural Experiment Station Clifford E. & Melda Snyder Research Farm, Pittstown, NJ. The field was prepared by disc plowing. The soil was fertilized with 1009 kg/ha of 15-15-15 N-P-K followed by addition of raised beds which were established mechanically. At last, a drip irrigation system was installed under the 0.032 mm plastic mulch. The plants were spaced 0.3 m apart within a 2.1 m plot and the plots were spaced 0.9 m apart within a row. The rows were placed 1.8 m apart from each other. The field was weeded mechanically in between rows and manually within the rows throughout the experiment. The weather variables during the time of the experiment are summarized in Table 4. 

### 3.2. Harvest and Post-Harvest Processing

In 2017, the plants were harvested twice, first on July 30th and then again roughly two months later, on 29th September. The plants were allowed to regrow in between the harvests and after the first harvest, the plants were cutback up to 10 cm above ground level. The same plants were allowed to overwinter in the field after the second harvest in 2017 and harvested again in 2018 on 28th June. The plants were harvested while they were in their full flowering stage during all 3 harvests. The harvested shoot biomass was dried at 37 °C in a walk-in forced air dryer by Powell Tobacco (MarCo Manufacturing Company LLC, Bennettsville, SC, USA) and weighed to determine the final dry mass.

### 3.3. Sample Preparation

The dried plant shoots were ground and then extracted. Approximately 100 mg of ground plant powder was extracted with 10 mL of ethanol and sonicated for 5 min in a Branson M5800 Ultrasonic Cleaner (Branson Ultrasonics, Brookfield, CT, USA). The samples were then allowed to stand at room temperature overnight. The extracts were then diluted 25 times by adding 40 μL of the extract to 960 μL of methanol and vortexed at full speed to homogenize the sample. The diluted extract was then centrifuged at 10,000× *g* for 10 min using the accuSpin Micro17 centrifuge (Fisher Sci, Hampton, NH, USA). An aliquot of 700 μL was transferred to an HPLC vial and stored at −20 °C until subsequent analysis.

The concentration of each compound was calculated using a calibration curve that was prepared by mixing the stock solutions of reference standards of Nepetalic Acid (NA), Nepetalactam (NT), Dihydronepetalactone (DHNL) [66]. The stock solution was prepared by mixing stock solutions of reference standards of nepetalic acid at ca 20 μg/mL and nepetalactam and dihydronepetalactone at ca 5 μg/mL. The stock solutions were diluted 11 times more by a 2-fold serial dilution. The concentration of the compounds was calculated using the calibration curve and is expressed as mg/100 g dry weight.

### 3.4. Instrumentation and Software

The Agilent 1290 Infinity II UHPLC in conjunction with the 6470 Triple Quadrupole Mass Spectrometer (QqQ-MS), featuring an electrospray ionization (ESI) source from Agilent Technologies (Santa Clara, CA, USA) was used for analyzing the samples. The column used was a Waters Acquity UPLC BEH C18, 2.1 × 50 mm, 1.7 μm (Milford, MA, USA). Agilent MassHunter Workstation Data Acquisition (version B.10.00), Qualitative Analysis (version B.07.00), and Quantitative Analysis (version B.07.01) were used for data acquisition and processing.

### 3.5. UHPLC-QqQ/MS Method

For the chromatographic separation of analytes, water with 0.1% formic acid was used as mobile phase A and acetonitrile with 0.1% formic acid as mobile phase B. The elu-tion gradient was 90% A and 10% B from 0 min to 0.2 min, 30% B from 0.2 min to 4 min, then held isocratically at 45% B from 4 min to 4.5 min and increased to 100% B from 4.6 min to 6 min. The column was washed with 100% B for 1 min and then the gradient was switched back to 10% B for 1 min for equilibrating the column before the next injection. The total runtime of the method was 7 min. The eluent before 1 min and after 4.5 min was eluted to the waste. The flow rate was 0.4 mL/min and the injection volume was 0.7 mL. Each injection was followed by a 3 s standard needle wash using 70% methanol. The column temperature was thermostatted at 30 °C. The autosampler temperature was maintained at 4 °C throughout the sample run.

For the QqQ-MS/MS parameters, high purity nitrogen was used as the nebulizing, sheath and drying gas. The drying gas was set at 300 °C with a flow rate of 13 L/min, the nebulizer at 50 psi and the sheath gas at 250 °C with a flow rate of 12 L/min. The capillary voltage was 3000 V and nozzle voltage was set at 1500 V. Delta EMV was zero. Multiple reaction Monitoring mode (MRM) was used as scanning mode with parameters shown in Table 5. Representative chromatograms of nepetalic acid (NA), nepetalactam (NT) and dihydronepetalactone (DHNL) along with their chemical structures are presented in Figure 9.

### 3.6. Statistical Analysis

The experimental design is a split plot in time scheme where the 34 genotypes are the plots, and the 3 harvesting times are subplots. The plots are structured in a randomized complete block design, with 3 blocks and 7 plants as an experimental unit. Analysis of variance (ANOVA) was conducted, and the means were compared by the Scott-Knott test, using the statistical software Assistat 7.7 [67].

## 4. Conclusions

While data on the effect of genotype and harvest time on nepetalic acid, nepetalactam, and dihydronepetalactone is scarce, the current study offers preliminary insight into the potential effect of genotype, harvest time and their interaction on biomass and secondary metabolites in catnip. In this study, the interaction effect of genotype and harvest time on the biomass and secondary metabolites is significant, showing the potential to explore new genotypes in different environments and growing conditions to increase the yield of compounds of interest. The hybrid CR9 × CR3, coming from the crossing of two recently patented cultivars, CR9 and CR3, shows remarkable potential for aboveground biomass production, although it is not as productive as productive as the parent CR9 in terms of secondary metabolites of interest. Genotype CR5 shows contents of nepetalic acid comparable to cultivar CR9 in two harvests and the highest accumulated yield of dihydronepetalactone and is also a potential genotype for economic exploration. Previously uncharacterized genotypes such as C245, CR2 and UK.15, despite not showing a particularly high yield of iridoid metabolites, can be used in breeding programs considering the high productivity of aboveground biomass. Genotype UK.2, characterized here for the first time, can be highlighted by the highest accumulation of nepetalactam among the studied genotypes. Genotypes such as CL1, CL2, UK.1, UK.13, UK.14 and UK.17 are among the plants that accumulated the least amount of iridoid metabolites. Such plants can be further screened for the production of other metabolites such as non-iridoid terpenes, considering the substantial chemodiversity observed in this species. The development of new and improved genetic materials is a fundamental aspect for establishing catnip as a reliable source of natural products for the insect repellent industries and other markets. Future studies should address the development of such materials as well as their interactions with specific agronomic practices.

## Figures and Tables

**Table 1:** Sum of square values for biomass, Nepetalic acid (NA), Nepetalactone (NT) and Dihydronepetalactone (DHNL).

Source of Variation	df	Sum of Square Values			
		Biomass	NA	NT	DHNL
Blocks	2	4048.9 ^ns^	315.0 *	891.8 **	11.2 ^ns^
Genotype (G)	33	104,207.0 **	16,069,898.7 **	7880.9 **	248.2 **
*Error I*	*66*	*55,961.39*	*1,052,239.5*	*3588.2*	*182.4*
Plot	101	164,217.3	17,122,453.3	12360.9	441.9
Harvest (H)	2	9662.9 *	4,399,046.6 **	20,016.4 **	541.9 **
Interaction GxH	66	186,209.2 **	6,155,087.9 **	5929.9 **	202.5 *
*Error II*	*136*	*180,248.7*	*1,496,172.6*	*5786.1*	*288.5*
**Total**	**305**	**540,338.17**	**29,172,760.4**	**44,093.2**	**1474.9**

* Statistically significant at 5% probability level; ** Statistically significant at 1% probability level; ^ns^ not significant; df: degrees of freedom.

**Table 2:** Average biomass yield and nepetalic acid (NA), nepetalactam (NT) and dihydronepetalactone (DHNL). content in catnip genotypes.

Genotype	Biomass (g/Plant)	NA (mg/100 g)	NT (mg/100 g)	DHNL (mg/100 g)
C243	116.9 a *	216.5 e	6.0 c	3.9 c
C244	115.1 a	236.1 e	7.3 c	4.2 c
C245	127.0 a	252.4 e	3.5 c	3.4 c
C246	86.8 b	142.7 e	5.6 c	3.7 c
C247	122.7 a	200.1 e	8.1 c	4.8 c
C248	119.7 a	161.1 e	7.6 c	3.8 c
CIT	100.8 b	200.3 e	2.8 c	4.0 c
CL1	112.7 a	11.9 f	4.5 c	3.8 c
CL2	98.9 b	12.4 f	4.2 c	3.8 c
CN3	111.4 a	361.5 d	10.8 c	4.8 c
CN5	114.3 a	575.5 c	9.8 c	4.5 c
CN6	130.9 a	111.8 f	8.6 c	4.5 c
CR1	119.8 a	566.7 c	19.1 b	5.8 b
CR2	132.5 a	564.0 c	14.5 b	6.5 b
CR2.3	104.7 b	542.8 c	15.2 b	6.0 b
CR3	119.8 a	327.8 d	13.9 b	4.5 c
CR5	121.4 a	708.4 b	16.9 b	7.9 a
CR9	142.1 a	863.6 a	19.1 b	5.7 b
CR9 × CR3	155.6 a	660.0 b	10.4 c	4.5 c
G1	104.0 b	198.0 e	8.7 c	4.3 c
UK.1	124.1 a	22.1 f	7.2 c	4.6 c
UK.2	134.4 a	538.3 c	25.5 a	5.5 b
UK.4	128.6 a	243.2 e	10.9 c	4.3 c
UK.5	72.6 c	293.3 d	11.7 c	4.3 c
UK.6	92.1 b	160.8 e	5.6 c	4.6 c
UK.7	126.6 a	213.5 e	8.8 c	4.4 c
UK.9	59.8 c	728.8 b	12.3 c	5.3 b
UK.10	113.9 a	217.1 e	8.6 c	4.2 c
UK.11	107.9 a	192.1 e	11.1 c	4.4 c
UK.12	108.7 a	213.2 e	6.1 c	4.5 c
UK.13	112.7 a	24.2 f	4.2 c	4.7 c
UK.14	109.5 a	23.8 f	5.8 c	4.7 c
UK.15	131.7 a	280.8 d	7.0 c	4.2 c
UK.17	97.7 b	24.2 f	4.1 c	3.7 c

* Means followed by the same letters in the columns do not differ statistically according to the Scott-Knott test at 5% probability level.

**Table 3:** Average biomass yield and nepetalic acid (NA), nepetalactam (NT) and dihydronepetalactone (DHNL) content of catnip genotypes content in each harvest.

Harvest	Biomass (g/Plant)	NA	NT	DHNL
			(mg/100 g)	
2017-1 (July 2017)	115.0 a *	262.8 b	21.0 a	6.4 a
2017-2 (September 2017)	120.4 a	169.8 c	3.7 b	3.2 c
2018 (June 2018)	106.7 b	457.6 a	4.1 b	4.4 b

* Means followed by the same letters in the columns do not differ statistically according to the Scott-Knott test at 5% probability level.

**Table 4:** Monthly averages of weather variables in Pittstown, NJ, USA from May 2017 to June 2018. Source: Njweather.org.

Month	Year	Precipitation (mm)	Temperature (°C)	Wind Speed (km/h)	Solar Radiation (W/m^2^)	Relative Humidity (%)	Dew Point (°C)
May	2017	164	14.7	9.3	150.9	75.6	9.3
June	2017	74	20.9	9.8	253.3	68.5	14.1
July	2017	189	22.9	7.6	237.8	76.6	18.2
August	2017	92	20.9	7.1	215.4	77.2	16.3
September	2017	484	19.0	7.6	176.5	77.4	14.4
October	2017	109	15.3	9.7	129.6	75.2	10.4
November	2017	33	5.9	10.5	93.2	71.3	0.72
December	2017	37	−0.7	10.8	67.8	70.4	−6.1
January	2018	61	−2.7	12.4	87.5	68.2	−8.5
February	2018	156	3.1	10.5	91.8	78.2	−0.9
March	2018	106	2.0	13.0	155.8	66.5	−4.2
April	2018	120	7.7	13.5	179.2	64.1	−0.4
May	2018	137	17.8	9.5	195.0	74.4	12.2
June	2018	61	20.5	9.2	246.8	71.4	14.2

**Table 5:** Multiple reaction monitoring (MRM) parameters for catnip bioactive compounds.

Compounds	RT (min)	Precursor (*m*/*z*)	Frag	Quant (*m*/*z*)	CE (eV)	Qual 1 (*m*/*z*)	CE (eV)	Ratio	Qual 2 (*m*/*z*)	CE (eV)	Ratio
NA (1 & 2)	1.68/1.95	167.0	65	81.1	13	123.1	5	0.59	79.0	29	0.40
NT	2.12	166.0	110	110.0	25	65.0	50	0.94	92.0	37	0.88
DHNL	2.85	169.0	80	123.1	9	81.1	21	0.48	79.1	37	0.10

For MRM abbreviations, RT = retention time; Frag = fragmentor voltage; Quant = quantifier ion; Qual = qualifier ion; CE = collision energy. Qualifier ions’ ratio is the peak area of qualifier ion divided by peak area of associated quantifier ion. NA = Nepetalic Acid, NT = Nepetalactam, DHNL = Dihydronepetalactone.

## Data Availability

Data associated with this study can be found as Supplementary Materials. Additional data are available on request from the corresponding author.

## Supplementary Materials

The following supporting information can be downloaded at: https://www.mdpi.com/article/10.3390/molecules27207057/s1, Table S1: Interaction between catnip genotypes and harvest times for dry biomass accumulation.; Table S2: Interaction between catnip genotypes and harvest times for Nepetalic Acid (NA) accumulation; Table S3: Interaction between catnip genotypes and harvest times for Nepetalactam (NT) accumulation; Table S4: Interaction between catnip genotypes and harvest times for Dihydronepetalactone (DHNL) accumulation; Table S5: Total accumulated yield of biomass (dry weight) and total content of compounds Nepetalic Acid (NA), Nepetalactam (NT) and Dihydronepetalactone (DHNL) per plant in 2017 and 2018.
