# Supplementary material for: Investigation of Volatile Iridoid Terpenes in Nepeta cataria L. (Catnip) Genotypes
Source: Molecules. 2022 Oct 19;27(20):7057. doi: 10.3390/molecules27207057 (PMC9607335; doi:10.3390/molecules27207057)
Supplement: Supplementary file 1 [file molecules-27-07057-s001.zip › molecules-1958994-supplementary.pdf]

Table S1. Interaction between catnip genotypes and harvest times for dry biomass accumulation.

Genotype	Harvest (g/plant) 2017-1	2017-2	2018
C243	131.0 aA	114.3 bA	105.6 aA
C244	90.5 aB	159.5 aA	95.3 bB
C245	185.7 aA	121.4 bB	73.8 bB
C246	59.5 aA	104.8 bA	96.0 bA
C247	100.0 aA	130.1 aA	138.1 aA
C248	121.4 aA	102.4 bA	135.4 aA
CIT	107.1 aA	95.2 bA	100.0 bA
CL1	147.6 aA	100.0 bA	90.5 bA
CL2	165.9 aA	100.0 bB	40.0 bC
CN3	121.4 aA	152.4 aA	60.5 bB
CN5	100.0 aA	114.3 bA	128.6 aA
CN6	131.0 aA	138.1 aA	123.8 aA
CR1	104.8 aA	147.6 aA	107.1 aA
CR2	104.8 aB	119.0 bB	173.8 aA
CR2.3	100.0 aA	142.7 aA	71.4 bA
CR3	97.6 aB	161.9 aA	100.0 bB
CR5	109.5 aA	100.0 bA	154.8 aA
CR9	102.4 aB	147.6 aA	176.2 aA
CR9xCR3	161.9 aA	116.7 bA	188.1 aA
G1	121.4 aA	111.9 bA	78.6 bA
UK.1	133.6 aA	102.4 bA	136.5 aA
UK.2	123.8 aA	147.6 aA	131.7 aA
UK.4	95.2 aB	169.0 aA	121.4 aB
UK.5	121.4 aA	57.1 bB	39.3 bB
UK.6	88.1 aA	104.8 bA	83.3 bA
UK.7	111.9 aA	142.9 aA	125.0 aA
UK.9	50.0 aA	76.2 bA	53.3 bA
UK.10	111.9 aA	103.6 bA	126.2 aA
UK.11	119.0 aA	147.6 aA	57.1 bB
UK.12	119.0 aA	97.6 bA	109.5 aA
UK.13	102.4 aA	104.8 bA	131.0 aA
UK.14	102.4 aA	111.9 bA	114.3 aA
UK.15	128.6 aA	131.0 aA	135.7 aA
UK.17	140.5 aA	116.7 bA	35.8 bB

Statistically significant differences among genotypes are indicated by lower-case letters and differences among the same genotype across harvests are indicated by upper-case letters. Means followed by the same letter do not differ according to the Scott-Knott test ($p \leq 0.01$).

Table S2. Interaction between catnip genotypes and harvest times for Nepetalic Acid (NA) accumulation.

	Harvest (mg/100g)		
**Genotype**	**2017-1**	**2017-2**	**2018**
C243	227.9 cA	143.7 bA	277.8 eA
C244	213.6 cA	156.9 bA	337.9 dA
C245	206.4 cB	180.0 bB	370.8 dA
C246	154.3 cA	124.1 bA	149.8 fA
C247	222.7 cA	140.4 bA	237.0 eA
C248	205.1 cA	129.8 bA	148.3 fA
CIT	41.5 dB	89.6 bB	469.7 dA
CL1	10.8 dA	5.0 bA	19.8 fA
CL2	14.7 dA	4.4 bA	18.1 fA
CN3	348.9 bA	307.6 aA	428.2 dA
CN5	587.4 aB	297.7 aC	841.4 cA
CN6	161.5 cA	80.3 bA	93.5 fA
CR1	439.5 bB	350.8 aB	909.6 cA
CR2	526.3 aB	248.4 aC	917.4 cA
CR2.3	420.5 bB	284.1 aB	923.6 cA
CR3	397.8 bA	171.1 bB	414.5 dA
CR5	565.8 aB	326.5 aC	1233.0 bA
CR9	675.9 aB	354.0 aC	1560.9 aA
CR9xCR3	454.8 bB	298.9 aB	1226.1 bA
G1	187.2 cA	133.1 bA	273.7 eA
UK.1	13.7 dA	13.3 bA	39.3 fA
UK.2	409.3 bB	369.7 aB	835.9 cA
UK.4	182.2 cA	194.4 bA	352.9 dA
UK.5	308.8 cA	158.6 bB	412.5 dA
UK.6	142.8 cA	141.2 bA	198.5 fA
UK.7	188.7 cA	182.2 bA	269.5 eA
UK.9	673.7 aB	232.3 aC	1280.4 bA
UK.10	230.2 cA	160.4 bA	260.8 eA
UK.11	212.5 cA	119.4 bA	244.4 eA
UK.12	201.2 cA	174.0 bA	264.5 eA
UK.13	14.6 dA	12.9 bA	45.1 fA
UK.14	13.7 dA	12.5 bA	45.1 fA
UK.15	258.3 cB	170.4 bB	413.7 dA
UK.17	22.0 dA	6.6 bA	44.0 fA

Statistically significant differences among genotypes are indicated by lower-case letters and differences among the same genotype across harvests are indicated by upper-case letters. Means followed by the same letter do not differ according to the Scott-Knott test ($p \leq 0.01$).

Table S3. Interaction between catnip genotypes and harvest times for Nepetalactam (NT) accumulation.

	Harvest (mg/100g)		
**Genotype**	**2017-1**	**2017-2**	**2018**
C243	11.8 dA	2.0 aA	4.3 aA
C244	15.9 cA	2.5 aB	3.5 aB
C245	5.1 dA	3.4 aA	2.0 aA
C246	13.4 dA	1.9 aB	1.5 aB
C247	20.5 cA	2.0 aB	1.7 aB
C248	20.1 cA	1.4 aB	1.2 aB
CIT	4.8 dA	1.8 aA	1.9 aA
CL1	11.3 dA	0.9 aA	1.4 aA
CL2	9.4 dA	1.2 aA	2.0 aA
CN3	21.1 cA	3.3 aB	8.1 aB
CN5	16.8 cA	8.6 aA	3.9 aA
CN6	22.2 cA	2.2 aB	1.5 aB
CR1	38.6 aA	8.6 aB	10.3 aB
CR2	29.6 bA	5.0 aB	8.8 aB
CR2.3	34.3 bA	7.2 aB	4.2 aB
CR3	36.5 bA	1.9 aB	3.4 aB
CR5	41.8 aA	5.9 aB	3.2 aB
CR9	40.5 aA	11.5 aB	5.2 aB
CR9xCR3	18.5 cA	6.5 aB	6.3 aB
G1	20.5 cA	2.8 aB	2.8 aB
UK.1	18.9 cA	1.0 aB	1.7 aB
UK.2	50.2 aA	9.1 aB	17.2 aB
UK.4	24.1 cA	4.2 aB	4.4 aB
UK.5	22.0 cA	2.7 aB	10.4 aB
UK.6	12.0 dA	3.1 aA	1.8 aA
UK.7	19.9 cA	3.6 aB	2.9 aB
UK.9	21.4 cA	5.8 aB	9.6 aB
UK.10	21.4 cA	2.8 aB	1.6 aB
UK.11	28.4 bA	2.4 aB	2.5 aB
UK.12	12.5 dA	3.1 aA	2.6 aA
UK.13	10.2 dA	1.2 aA	1.3 aA
UK.14	14.8 dA	1.2 aB	1.4 aB
UK.15	16.4 cA	2.6 aB	2.0 aB
UK.17	9.6 dA	1.4 aA	1.4 aA

Statistically significant differences among genotypes are indicated by lower-case letters and differences among the same genotype across harvests are indicated by upper-case letters. Means followed by the same letter do not differ according to the Scott-Knott test ($p \leq 0.01$).

Table S4. Interaction between catnip genotypes and harvest times for Dihydronepetalactone (DHNL) accumulation.

	Harvest (mg/100g)		
Genotype	2017-1	2017-2	2018
C243	5.1 cA	2.9 aA	3.7 aA
C244	6.0 cA	2.9 aB	3.7 aB
C245	3.9 cA	2.9 aA	3.4 aA
C246	5.3 cA	2.5 aB	3.3 aB
C247	7.3 cA	2.8 aB	4.2 aB
C248	5.4 cA	2.8 aB	3.1 aB
CIT	4.0 cA	3.3 aA	4.7 aA
CL1	5.3 cA	2.6 aA	3.4 aA
CL2	5.6 cA	2.7 aB	3.1 aB
CN3	6.4 cA	4.3 aB	3.6 aB
CN5	6.0 cA	3.3 aA	4.3 aA
CN6	6.9 cA	3.1 aB	3.3 aB
CR1	8.9 bA	3.5 aB	4.9 aB
CR2	11.2 aA	3.5 aB	4.9 aB
CR2.3	10.3 aA	3.3 aB	4.5 aB
CR3	5.0 cA	3.5 aA	5.0 aA
CR5	12.1 aA	4.1 aC	7.5 aB
CR9	7.0 cA	4.6 aA	5.7 aA
CR9xCR3	4.7 cA	4.2 aA	4.4 aA
G1	6.1 cA	3.0 aB	4.2 aB
UK.1	6.3 cA	3.3 aB	4.2 aB
UK.2	8.3 bA	3.3 aB	4.8 aB
UK.4	5.7 cA	2.5 aB	4.6 aA
UK.5	5.8 cA	2.4 aB	4.7 aA
UK.6	7.0 cA	3.4 aB	3.4 aB
UK.7	5.9 cA	2.9 aA	4.5 aA
UK.9	6.4 cA	3.4 aB	6.2 aA
UK.10	6.0 cA	2.8 aB	3.9 aB
UK.11	5.9 cA	2.7 aB	4.7 aA
UK.12	6.3 cA	3.3 aB	3.8 aB
UK.13	6.3 cA	3.2 aB	4.5 aB
UK.14	5.5 cA	3.5 aA	5.2 aA
UK.15	5.4 cA	2.6 aA	4.5 aA
UK.17	4.3 cA	2.6 aA	4.2 aA

Statistically significant differences among genotypes are indicated by lower-case letters and differences among the same genotype across harvests are indicated by upper-case letters. Means followed by the same letter do not differ according to the Scott-Knott test ($p \leq 0.05$).

Table S5. Total accumulated yield of biomass (dry weight) and total content of compounds Nepetalic Acid (NA), Nepetalactam (NT) and Dihydronepetalactone (DHNL) per plant in 2017 and 2018**.**

Genotype	Biomass **(g/plant)**			NA **mg/plant**			NT			DHNL		
	2017	*2018*	***2017+ 2018***	*2017*	*2018*	***2017+ 2018***	*2017*	*2018*	***2017+ 2018***	*2017*	*2018*	***2017+ 2018***
C243	209.0	105.6	**314.5**	410.5	293.3	**703.8**	17.0	4.5	**21.5**	8.9	3.9	**12.8**
C244	146.6	95.3	**241.9**	281.4	321.8	**603.2**	15.7	3.4	**19.1**	7.0	3.5	**10.5**
C245	265.1	73.8	**338.9**	516.1	273.7	**789.8**	11.6	1.5	**13.0**	9.2	2.5	**11.7**
C246	174.1	96.0	**270.1**	244.7	143.8	**388.5**	14.2	1.4	**15.6**	7.0	3.2	**10.2**
C247	168.9	138.1	**307.0**	319.5	327.3	**646.9**	21.9	2.4	**24.2**	9.3	5.7	**15.0**
C248	188.3	135.4	**323.7**	335.9	200.8	**536.7**	25.3	1.7	**27.0**	8.4	4.3	**12.6**
CIT	166.9	100.0	**266.9**	98.0	469.7	**567.7**	6.2	1.9	**8.1**	6.3	4.7	**11.0**
CL1	232.3	90.5	**322.8**	20.2	17.9	**38.1**	17.5	1.2	**18.7**	10.1	3.0	**13.1**
CL2	257.2	31.0	**288.2**	26.6	5.6	**32.2**	15.2	0.6	**15.8**	11.3	1.0	**12.3**
CN3	195.0	60.5	**255.5**	650.0	258.9	**908.9**	28.1	4.9	**32.9**	11.0	2.2	**13.2**
CN5	214.3	128.6	**342.9**	927.6	1081.8	**2009.4**	26.7	5.1	**31.7**	9.8	5.6	**15.3**
CN6	209.9	123.8	**333.8**	274.9	115.7	**390.7**	30.8	1.8	**32.7**	11.5	4.1	**15.6**
CR1	195.3	107.1	**302.5**	778.2	974.6	**1752.8**	48.2	11.0	**59.2**	12.5	5.2	**17.7**
CR2	196.3	173.8	**370.1**	778.8	1594.5	**2373.3**	35.6	15.3	**51.0**	15.0	8.5	**23.5**
CR2.3	157.1	71.4	**228.6**	582.9	659.7	**1242.5**	38.4	3.0	**41.4**	12.2	3.2	**15.4**
CR3	167.4	100.0	**267.4**	507.7	414.5	**922.2**	36.9	3.4	**40.3**	7.3	5.0	**12.3**
CR5	171.7	154.8	**326.5**	822.7	1908.2	**2730.9**	49.4	4.9	**54.4**	15.8	11.6	**27.4**
CR9	157.7	176.2	**333.9**	887.8	2750.2	**3638.0**	47.8	9.1	**56.9**	9.7	10.0	**19.7**
CR9xCR3	275.1	188.1	**463.2**	1074.7	2306.3	**3380.9**	69.7	11.8	**49.1**	12.4	8.4	**20.8**
G1	205.6	78.6	**284.1**	339.3	215.1	**554.4**	54.4	2.2	**29.5**	9.9	3.3	**13.2**
UK.1	188.6	136.5	**325.1**	25.5	53.7	**79.2**	46.9	2.3	**23.4**	9.5	5.8	**15.2**
UK.2	191.2	131.7	**322.9**	755.8	1101.1	**1856.8**	160.9	22.7	**90.9**	12.6	6.3	**18.9**
UK.4	160.5	121.4	**281.9**	300.4	428.6	**728.9**	74.5	5.3	**31.0**	7.1	5.5	**12.6**
UK.5	218.4	39.3	**257.7**	528.7	162.1	**690.8**	44.1	4.1	**33.4**	9.4	1.8	**11.3**
UK.6	144.7	83.3	**228.0**	205.7	165.4	**371.1**	29.1	1.5	**13.8**	8.1	2.9	**10.9**
UK.7	179.3	125.0	**304.3**	334.1	336.9	**671.0**	59.9	3.7	**28.3**	8.5	5.6	**14.1**
UK.9	78.6	53.3	**131.9**	403.2	682.9	**1086.1**	34.5	5.1	**17.5**	4.2	3.3	**7.5**
UK.10	200.3	126.2	**326.5**	399.3	329.1	**728.4**	52.2	2.1	**28.5**	9.2	4.9	**14.1**
UK.11	214.5	57.1	**271.7**	367.0	139.6	**506.7**	82.4	1.4	**37.6**	9.6	2.7	**12.3**
UK.12	186.0	109.5	**295.5**	356.0	289.7	**645.7**	33.8	2.8	**19.8**	9.8	4.1	**13.9**
UK.13	161.0	131.0	**292.0**	22.5	59.0	**81.6**	23.4	1.7	**12.9**	8.3	6.0	**14.3**
UK.14	183.9	114.3	**298.2**	24.3	51.6	**75.8**	34.3	1.6	**17.7**	8.5	5.9	**14.4**
UK.15	196.4	135.7	**332.1**	447.7	561.4	**1009.1**	49.0	2.6	**25.4**	8.7	6.1	**14.8**
UK.17	226.8	35.8	**262.7**	36.5	15.8	**52.3**	28.0	0.5	**15.1**	8.3	1.5	**9.8**
